# Sweat evaporation in humans: A molecular and thermodynamic perspective

**DOI:** 10.1113/EP093011

**Published:** 2025-07-28

**Authors:** Edward T. Ashworth

**Affiliations:** ^1^ Faculty of Medicine and Health University of Sydney Sydney New South Wales Australia

**Keywords:** heat exchange, sweat physiology, thermodynamics, thermoregulation

## Abstract

Evaporative heat loss through sweating is essential for maintaining thermal balance in humans, particularly during exercise or in hot environments. Although the physiological mechanisms regulating sweat production and skin blood flow are well documented, the molecular processes underpinning sweat evaporation are less often considered. This review explores the physics of sweat evaporation from first principles, examining how energy is transferred, how water molecules escape the liquid phase and how this process is shaped by local and systemic factors. At the molecular level, evaporation occurs when surface water molecules attain sufficient kinetic energy to overcome hydrogen bonding. The energy required for this phase change, the latent heat of vaporisation, is supplied via conduction from the skin and, ultimately, from core body heat. The molecular energy within the sweat layer follows a Boltzmann distribution, meaning that only a subset of molecules have sufficient energy to evaporate at any time. As these high‐energy molecules escape, the remaining sweat cools, helping to lower body temperature. This process continues as long as heat is resupplied via skin blood flow. Environmental conditions, such as humidity, airflow and clothing, affect the likelihood that evaporated molecules will remain in the vapour phase, while electrolytes in sweat can slightly reduce vapour pressure by locally altering the bonding structure of water. These factors determine how effectively sweat can evaporate by influencing surface area and liquid retention. By linking classical thermodynamics to human physiology, this review presents a unified framework for understanding how molecular interactions, statistical physics and environmental conditions converge to influence heat loss.

## Abstract (highlights)

- **What is the topic of this review?**
  This review examines the molecular and thermodynamic mechanisms underpinning sweat evaporation in humans, linking them to physiological function and environmental influences on heat loss.
- **What advances does it highlight?**
  It highlights that evaporative cooling is governed by probabilistic molecular interactions and energy distributions, rather than sweat volume alone. This reframing improves understanding of thermophysiology and informs more targeted strategies for managing thermal strain in health, performance and environmental adaptation.

## 1 INTRODUCTION

Evaporative cooling through sweating is essential for human thermoregulation, particularly during physical exertion and in warm environments (Cramer et al., 2022). Although the physiological mechanisms controlling sweat production and skin blood flow are fairly well understood, the molecular processes that govern how sweat removes heat are often underappreciated. Heat loss during evaporation is not merely the result of liquid water disappearing from the skin but reflects deeper physical principles involving molecular energy distributions, phase changes and the statistical behaviour of particles.

At the molecular level, evaporation occurs when individual water molecules (H_2_O) at the surface of the skin gain enough energy to overcome the cohesive forces that bind them to neighbouring molecules in the liquid phase (Bazrafshan et al., 2018; Luiten et al., 1996; Tabe et al., 2022). This energy‐dependent process is influenced not only by the temperature of the skin but also by the statistical distribution of molecular energies within a sweat droplet (Luiten et al., 1996; Sandomirskiy & Tamuz, 2023). Understanding how temperature, molecular motion, electrolyte concentrations and surface properties interact to influence evaporation provides deeper insight into why physiological mechanisms, such as increased skin blood flow and sweat rate, effectively support heat loss. This review aims to bridge the gap between classical physiological understanding and the underlying physics of sweat evaporation. It begins with an examination of the molecular mechanics of sweat evaporation, then links these processes to thermodynamic principles, and finally considers how human physiology regulates them to maintain thermal balance.

## 2 HEAT AS MOLECULAR ENERGY AND ITS PHYSIOLOGICAL CONSEQUENCES

In scientific terms, heat is not a substance but a form of energy (Sullivan & Spencer, 2022). Temperature reflects the average kinetic energy of a group of molecules, encompassing their translational, rotational and vibrational motion (Sullivan & Spencer, 2022). Therefore, when heat is transferred to molecules, their motion increases. This energy can be exchanged between molecules, raising or lowering the internal energy of a system. In the body, such energy transfer plays a fundamental role in driving chemical processes, with higher molecular energy increasing reactivity (Tansey & Johnson, 2015). Therefore, maintaining body temperature within a range that supports an optimal number of biochemical reactions is critical for cellular and systemic function.

When heat levels rise too high, this balance is disrupted. Excess heat introduces uncontrolled molecular energy into systems that rely on precise structural organisation and tightly regulated reaction rates. As temperature rises, increased molecular motion can destabilise these systems in several ways (Cramer et al., 2022; Roti Roti, 2008). Proteins, which rely on specific three‐dimensional structures to function, are particularly vulnerable. Increased kinetic energy can disrupt the hydrogen bonds and other weak interactions that maintain protein folding, leading to misfolding or denaturation (Roti Roti, 2008). Lipid bilayers in cell membranes also become excessively fluid, compromising membrane integrity and disrupting the function of ion channels and embedded proteins (Fan & Evans, 2015). Enzymatic reactions might accelerate unpredictably at high temperatures, losing their coordination and control (Cramer et al., 2022). This biochemical instability can lead to an overproduction of reactive oxygen species, which damage DNA, proteins and membranes (Belhadj Slimen et al., 2014).

It is not that high‐energy molecules are inherently toxic, but that when too many molecules acquire excess kinetic energy, the ordered complexity of biological systems is disrupted. The removal of excess heat from the body is therefore essential to maintaining physiological stability, and this process is achieved primarily through sweat evaporation (Cramer et al., 2022).

## 3 THE MOLECULAR BASIS OF SWEAT LOSS

Sweat production by eccrine glands forms the foundation of human evaporative cooling (Taylor & Machado‐Moreira, 2013). Composed predominantly of water (∼99%) with small amounts of electrolytes, such as sodium and chloride (Lara et al., 2016), sweat originates from plasma and interstitial fluid. It is presumed to be secreted at or near skin temperature, because thermal equilibration is likely to occur during its passage through the sweat duct. However, direct measurement of sweat temperature at the point of secretion remains limited by methodological challenges (Jaiswal et al., 2024). Upon reaching the skin surface, sweat forms a film whose structure is influenced not only by its temperature but also by the molecular interactions between water molecules.

**FIGURE 1:**
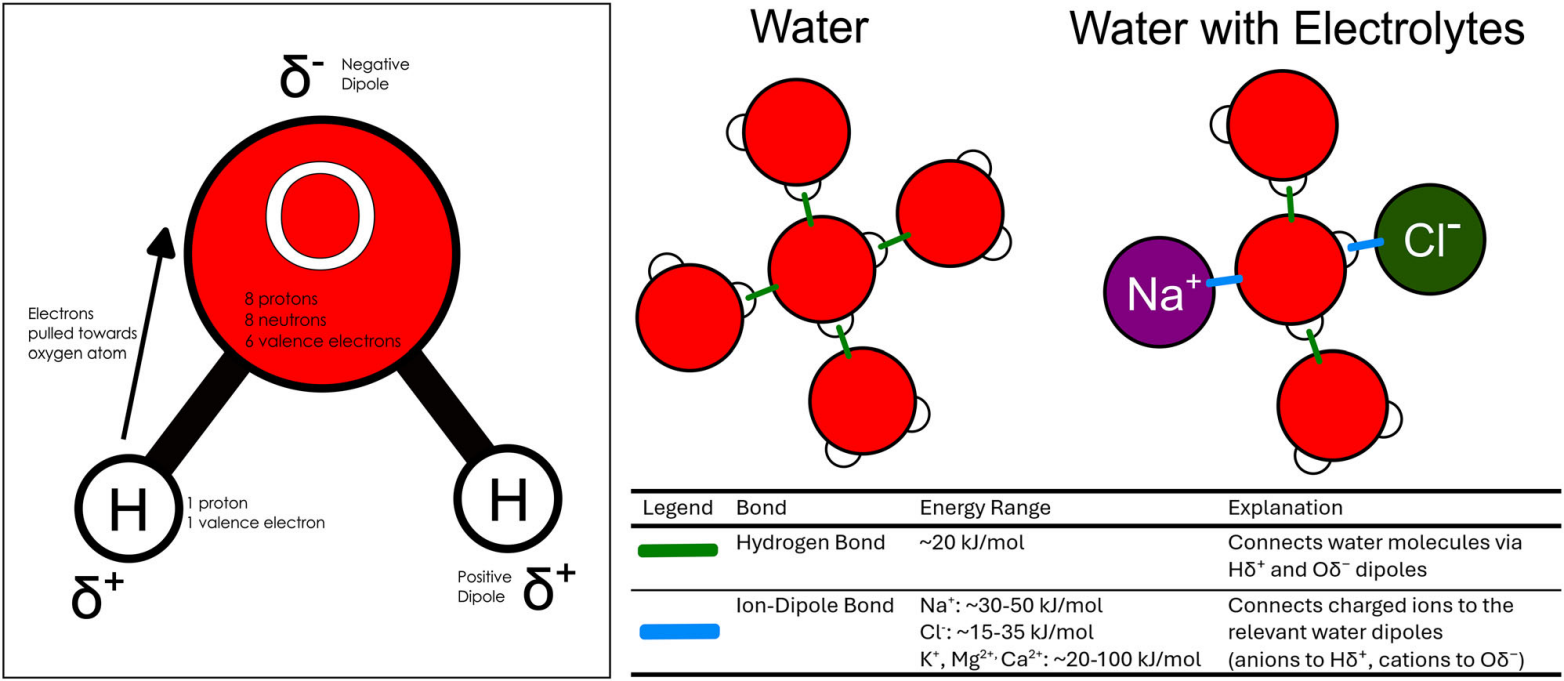
Molecular structure of water and the interactions of water molecules. The uneven electron distribution in water creates permanent dipoles, which are regions with partial positive and negative charges that enable hydrogen bonding between molecules. This cohesive network is locally disrupted by electrolytes, which form stronger ion–dipole interactions with water and alter its bonding structure and evaporative behaviour. The number of ion–dipole interactions shown is illustrative and represents an exaggerated scenario to highlight their local effects.

Within the sweat, water molecules are held together by hydrogen bonds. These are a type of intermolecular force formed between adjacent water molecules, specifically between the partly positive hydrogen atom of one molecule and the partly negative oxygen atom of another (Figure 1). These partial charges are referred to as dipoles (Debye, 1913). They arise because oxygen is more electronegative than hydrogen (Brini et al., 2017), owing primarily to its greater number of positively charged protons in the nucleus (Figure 1). These charged protons exert a stronger nuclear attractive force on the electrons in the covalent bond, pulling them closer to the oxygen atom. As a result, the oxygen atom carries a slight negative charge (δ^−^), while each hydrogen carries a slight positive charge (δ^+^) (Brini et al., 2017). When the positive dipole of a hydrogen atom in one water molecule is near the negative dipole of an oxygen atom in an adjacent molecule, an electrostatic attraction occurs; this is the hydrogen bond (Figure 1) (Brini et al., 2017). Hydrogen bonds are relatively strong in comparison to other intermolecular forces, contributing to the high cohesion and surface tension of water (Huš & Urbic, 2012).

Each water molecule possesses kinetic energy, reflected in its constant vibration, rotation and translational motion (Sullivan & Spencer, 2022). For evaporation to occur, this kinetic energy must reach the threshold required to overcome the cohesive hydrogen bonds and surface tension that keep the molecule in the liquid phase (Musolino & Trout, 2013). To achieve this, the molecule must absorb a fixed quantity of energy known as the latent heat of vaporisation (Cramer et al., 2022). For water, this is remarkably high, ∼2426 J g^−1^ (Wenger, 1972), meaning that each evaporated water molecule removes a substantial amount of energy from the body. This estimate, originally derived from physiological data (Wenger, 1972), has been consistently supported and refined by modern thermodynamic models, which report ∼2430 J g^−1^ at 30°C and 2415 J g^−1^ at 37°C (Wagner & Pruß, 2002), demonstrating minimal variation across the physiological temperature range. This energy is consumed during the phase change, both to break intermolecular bonds and to supply the kinetic energy required for the molecule to exist in the gas phase. As high‐energy molecules escape, the average kinetic energy of the remaining sweat decreases, producing the cooling effect fundamental to thermoregulation during exercise and heat stress. A molecular‐level understanding of this process, governed by energy thresholds and intermolecular forces, provides a clearer explanation of how the body dissipates heat and maintains thermal homeostasis.

**FIGURE 2:**
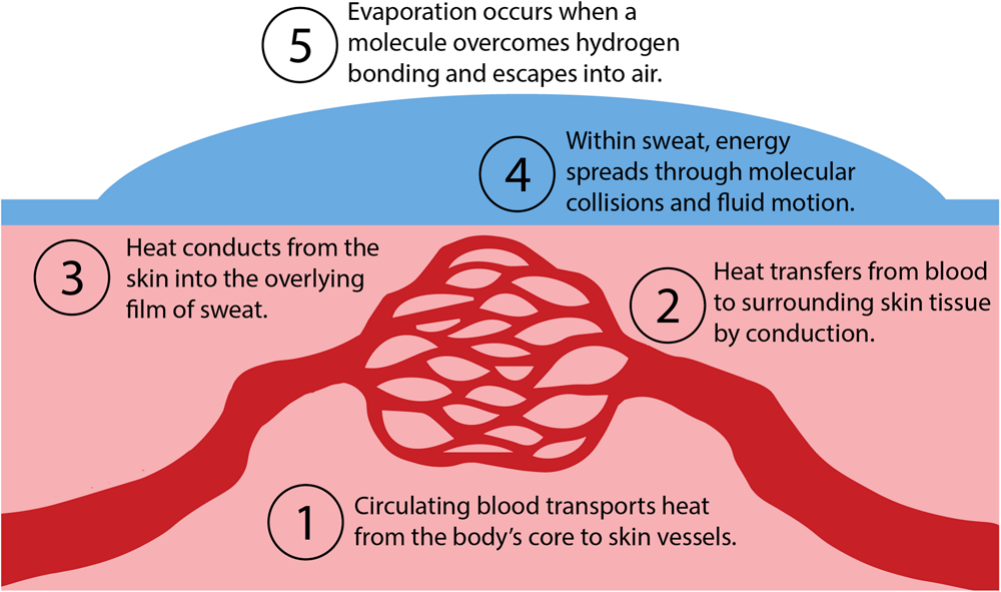
Schematic representation of heat transfer from the body core to the environment through the evaporation of sweat.

After sweat has formed on the skin, it must absorb additional thermal energy from the body in order to evaporate. This energy is transferred primarily by conduction, via the direct movement of heat from the warmer skin tissue into the cooler sweat layer in contact with it (Figure 2; Tansey & Johnson, 2015). At the molecular level, this involves collisions between the faster‐moving, higher‐energy molecules in the skin and the slower‐moving molecules in the overlying sweat (Wang et al., 2012). As these interactions occur, kinetic energy is transferred into and through the sweat, raising the energy of individual water molecules (Figure 2). Heat can then be transferred throughout the sweat through molecular collisions that transfer kinetic energy, or through the collective movement of fluid parcels containing higher‐energy molecules (Khan & Alzahrani, 2020). When molecules move as part of a fluid parcel, they do so while remaining loosely associated through hydrogen bonds, allowing them to move collectively. This bulk movement is driven by differences in kinetic energy whereby warmer, more energetic molecules spread apart, reducing the density of the parcel and causing it to rise relative to cooler, denser regions (Wang, 2024). The heat flow is sustained by the circulation of warm, core‐temperature blood, with thermal energy conducted through the extravascular tissue before reaching the skin surface (Charkoudian, 2003). As long as a temperature gradient exists between the skin and the sweat film, heat will continue to move into the sweat. This gradient is maintained on one side by the delivery of warm blood to the skin and on the other by evaporative cooling, which removes energy from the sweat layer and helps to keep it cooler than the underlying tissue.

Water is also lost from the skin through a passive process known as transepidermal water loss, whereby water diffuses through the lipid matrix of the outer skin layers. This process is continuous and unregulated but still produces fluid (∼300–500 mL day^−1^) that can evaporate from the skin surface (Gabard & Treffel, 2017). Its primary function is to support skin hydration and maintain barrier integrity, and in most conditions, it contributes negligibly to thermoregulatory heat loss (Gabard & Treffel, 2017). In contrast, sweat production by eccrine glands is an active process initiated specifically to dissipate heat. The much greater volume of fluid moved onto the skin surface during sweating (>1.5 L h^−1^) makes it the dominant mechanism for evaporative heat loss in humans (Gagnon & Crandall, 2018).

## 4 EQUATIONS THAT GOVERN EVAPORATIVE HEAT LOSS

Although the processes underlying sweat evaporation might appear complex and interdependent, they can be expressed using a set of simple equations that clearly illustrate the key factors influencing heat loss. The total energy lost from sweat evaporation ($Q_{\mathrm{evap}}$​) is equal to the mass of water that evaporates ($m_{\mathrm{evap}}$​) multiplied by the latent heat of vaporisation ($E_{\mathrm{loss}}$) (Cramer & Jay, 2019):

$$Q_{\mathrm{evap}}=m_{\mathrm{evap}}\times E_{\mathrm{loss}}$$

The latent heat of vaporisation is a temperature‐dependent physical constant (∼ 2426 J g^−1^) (Wenger, 1972). The mass of water that evaporates can be broken down further into the number of water molecules at the sweat–air interface ($N_{\mathrm{sweat}}$​), the probability that a molecule has sufficient energy to evaporate [$P(E\geq E_{\mathrm{vap}})$] (Tabe et al., 2022), the probability that an evaporated molecule remains in the vapour phase without recondensing ($f_{\mathrm{escape}}$​) (Bond & Struchtrup, 2004) and the mass of a single water molecule ($m_{H_{2}O}$):

$$m_{\mathrm{evap}}=N_{\mathrm{sweat}}\times P\left(E\geq E_{\mathrm{vap}}\right)\times f_{\mathrm{escape}}\times m_{H_{2}O}$$

The number of molecules at the sweat–air interface is proportional to the exposed skin surface area ($A_{\mathrm{skin}}$), the molecular density of the sweat layer ($\rho_{\mathrm{film}}$) and the depth of the evaporative layer (δ), which represents only the thin surface layer of molecules available for evaporation:

$$N_{\mathrm{sweat}}\propto A_{\mathrm{skin}}\times \rho_{\mathrm{film}}\times \delta$$

This formulation shows that individuals with a larger skin surface area can lose more heat through evaporation. However, they might also gain more heat from the environment in certain conditions, reflecting the balance required for thermal regulation.

## 5 THE ROLE OF THE BOLTZMANN CONSTANT AND BOLTZMANN DISTRIBUTION

**FIGURE 3:**
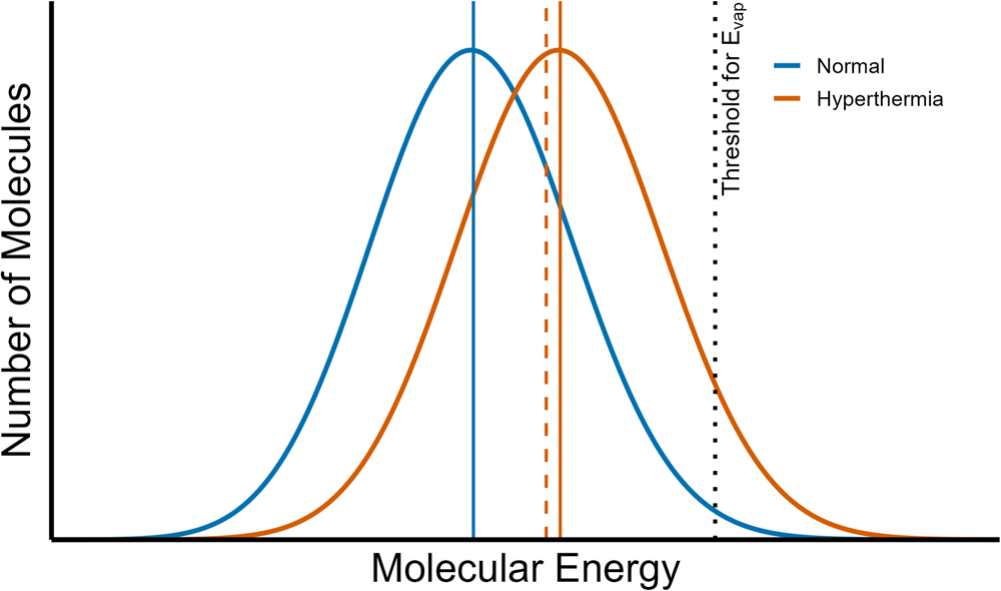
Boltzmann distributions of molecular energy in sweat in normal and hyperthermic conditions. Each curve represents the distribution of kinetic energies among water molecules in a thin layer of sweat. The hyperthermic condition shifts the distribution to the right, indicating a greater proportion of high‐energy molecules capable of evaporation. Mean molecular energies for each condition are shown as continuous vertical lines. The dotted black line marks the approximate energy threshold required for phase change ($E_{\mathrm{vap}}$). The dashed orange line shows the postevaporation mean energy in the hyperthermic conditions, assuming that all molecules above the evaporation threshold have transitioned to the vapour phase, illustrating the cooling effect of high‐energy molecule loss. Following evaporation, energy is redistributed among the remaining molecules, allowing the process to continue. This conceptual model illustrates how increased temperature enhances evaporative cooling through the statistical distribution of molecular energy.

The second factor in the equation, $P(E\geq E_{\mathrm{vap}})$, reflects the probability of a molecule evaporating; specifically, that the energy (E) of the molecule is at or above the energy required to evaporate ($E_{\mathrm{vap}}$). Critically, this highlights that evaporation is a statistical process. One of the most interesting aspects of sweat evaporation is that it occurs even though skin temperature is well below the boiling point of water. This is possible because temperature represents an average value, not a fixed amount of energy possessed by every molecule. In reality, the kinetic energies of individual water molecules within a single drop of sweat are distributed across a wide range, known as the Boltzmann distribution (Figure 3).

The Boltzmann distribution, named after physicist Ludwig Boltzmann, describes how molecular energies are spread at any given temperature (Boltzmann, 1877; Luiten et al., 1996). Most molecules have moderate energy, while a small but important fraction possess much higher energies (Luiten et al., 1996; Sandomirskiy & Tamuz, 2023). These high‐energy molecules that exceed the threshold for vaporisation (Figure 3) are the ones that escape from the liquid into the air, driving the cooling process. The Boltzmann constant ($k_{B}=1.38\times 10^{-23}\;J\;\;K^{-1}$​) provides the link between the macroscopic measurement of temperature and the microscopic behaviour of individual molecules, allowing us to quantify how thermal energy is partitioned at the molecular level (Boltzmann, 1877). Without this natural statistical spread of energies, evaporative cooling would be far less effective in maintaining thermal balance.

This distribution of molecular energies is not static; rather, it is continuously regenerated through the ongoing exchange of energy between molecules in the liquid (Sandomirskiy & Tamuz, 2023). As high‐energy molecules at the surface evaporate, they remove thermal energy from the system, lowering the average kinetic energy and therefore the temperature of the remaining sweat (Figure 3). However, collisions between water molecules within the liquid rapidly redistribute energy, with some gaining kinetic energy while others lose it (Tabe et al., 2022). These collisions allow heat to be transferred to molecules at the surface, which may then evaporate. This constant microscopic jostling re‐establishes the Boltzmann distribution at the new, slightly lower temperature. As long as there is a source of heat (typically, from blood flow to the skin), this energy can replenish the system (Charkoudian, 2003), allowing more molecules to reach escape energy and sustain the evaporation process (Sandomirskiy & Tamuz, 2023). In this way, the sweat film operates as a thermodynamic system in constant flux that always tends towards equilibrium yet is constantly perturbed by evaporation and heat supply.

At the molecular level, the Boltzmann distribution describes how energy is partitioned statistically among particles in a system. It shows that at any given temperature, most molecules have moderate energy, while fewer possess the high energies needed for phase change. The Boltzmann factor is the mathematical expression that governs this distribution (Boltzmann, 1877), and is given by:

Boltzmannfactor=e−E−EkBTkBT

This exponential term expresses the probability that a single molecule will have energy (E) at a given temperature (T) in degrees kelvin (Boltzmann, 1877; Sandomirskiy & Tamuz, 2023). The Boltzmann constant ($k_{B}$) links the microscopic scale of molecular energy to the macroscopic property of temperature. As the temperature increases, the distribution shifts, increasing the proportion of molecules with sufficient energy to overcome intermolecular forces and evaporate. In this way, the Boltzmann factor connects molecular dynamics directly to observable thermal behaviour (Planck, 1901).

To apply this to sweat evaporation, the specific energy required for evaporation ($E_{\mathrm{vap}}$) is substituted in:

PE≥Evap∝e−Evap−EvapkBTkBT

This expression represents the fraction of molecules that have sufficient kinetic energy to overcome the hydrogen bonding and surface tension holding them in the liquid phase.

## 6 PHYSIOLOGICAL MODULATORS OF SWEAT EVAPORATION

Although sweat evaporation is fundamentally a molecular process driven by the kinetic energy of water molecules, its effectiveness in real‐world conditions is strongly modulated by both physiological and environmental factors (Cramer & Jay, 2019). Several interacting elements determine how much heat the body can lose through evaporation at any given time. Skin wettedness, or the amount of skin surface covered by sweat, plays a key role by influencing the available area for evaporation and the ability of sweat to remain as a thin film rather than dripping off. Electrolyte concentration in sweat slightly alters its vapour pressure and affects how easily water molecules can escape into the air. Skin blood flow regulates skin temperature by delivering core heat to the surface, maintaining the gradient needed for continuous evaporation.

Environmental conditions, particularly ambient humidity and airflow, alter the vapour pressure gradient between sweat and the surrounding air, which determines how quickly sweat can evaporate. Clothing and behaviour, such as the use of breathable fabrics, seeking shade or adjusting exercise intensity, can also influence the ability of the body to sustain effective evaporative cooling. Together, these factors interact to either enhance or limit how much thermal energy the body can dissipate through sweat, especially during exercise, heat exposure or hot working conditions.

### 6.1 Skin wettedness

Skin wettedness is the proportion of skin covered with sweat and is a primary factor in determining the capacity of the body for evaporative heat loss (Gagge, 1937). When sweat spreads into a thin, continuous layer over the skin, it creates an extensive surface area for water molecules to escape into the air. This thin film maximises evaporative efficiency by exposing more molecules directly to the surrounding environment. However, when sweat production exceeds the capacity for evaporation, sweat begins to pool and drip from the skin. In this state, a significant fraction of secreted sweat is lost without contributing to cooling, because it falls away before absorbing the latent heat of vaporisation. In everyday life, mild to moderate sweating during light activity often maintains a thin, even layer of moisture on the skin, allowing for efficient evaporation. In contrast, during heavy exertion or in humid conditions, sweat can be produced faster than it can evaporate, causing it to accumulate and drip off the skin, thereby reducing overall cooling efficiency (Ashworth et al., 2020). Physiological and behavioural strategies that preserve thin, spread‐out sweat, such as appropriate pacing, clothing choices and environmental adaptation, help to maintain effective thermoregulation.

The behaviour of sweat on the skin is governed by the balance between cohesive and adhesive forces at the molecular level (Bazrafshan et al., 2018). Water molecules are highly cohesive owing to hydrogen bonding, which causes them to cluster together (Huš & Urbic, 2012). However, when sweat is secreted onto the skin, adhesive forces between the water molecules and the skin surface encourage the formation of a continuous layer rather than discrete droplets (Rosenberg et al., 1973). Properties of the skin itself, such as texture, oiliness, hydration and microstructure, influence how effectively sweat can maintain this uniform coating (Elkhyat et al., 2017). Surface oils, such as sebum, form a hydrolipidic film that helps to retain moisture on the skin by impeding the evaporation of sweat. This film is composed of hydrophobic lipids that disrupt the hydrogen bonding network in sweat, reducing the likelihood that individual water molecules will acquire enough energy to escape into the vapour phase (Porter, 2001). Hair can limit evaporative efficiency further by reducing airflow across the skin and increasing local humidity, both of which diminish the vapour pressure gradient required for evaporation (Coelho et al., 2010). In conditions of high humidity or excessive sweat rates, the sweat layer can break down, pulling sweat into larger droplets that are more likely to drip off without evaporating (Gagnon & Crandall, 2018). It is possible, although uninvestigated, that hair could help to prevent this dripping and thereby promote sweat evaporation in more humid conditions. Conversely, in environments where evaporation is rapid, such as dry or windy conditions, the sweat layer is continuously replenished and efficiently cooled. This shift from a thin film to droplet formation can indicate when evaporative cooling becomes less efficient, emphasizing the importance of maintaining a balance between sweat production, evaporation rate and environmental conditions to optimise heat loss.

### 6.2 Electrolyte concentrations

Although sweat contains electrolytes, their effect on evaporation is minor. In comparison to pure water, sweat has a slightly lower vapour pressure and a marginally higher heat of evaporation owing to the presence of dissolved ions, primarily sodium and chloride ions derived from blood plasma (Baker et al., 2019). These dissolved ions interact with nearby water molecules through ion–dipole forces (Hribar et al., 2002; Urbic, 2014), which are typically stronger than hydrogen bonds, often exceeding 50 kJ mol^−1^ (Urbic, 2014), compared with ∼20 kJ mol^−1^ for hydrogen bonding between water molecules (Figure 1; Huš & Urbic, 2012). These interactions create tightly bound hydration shells around the ions, locally disrupting the extensive hydrogen bonding network (Hribar et al., 2002). This makes it marginally more difficult for some individual water molecules to escape into the vapour phase (i.e., it reduces $f_{\mathrm{escape}}$ via humidity). As a result, the vapour pressure of sweat is slightly lower than that of pure water, and the energy required for evaporation ($E_{\mathrm{vap}}$) increases, meaning that sweat with a higher electrolyte concentration evaporates somewhat less readily. Because only a small fraction of water molecules is directly involved in ion–dipole interactions at any given time (Lara et al., 2016), the overall effect on sweat evaporation is subtle and likely to be negligible in most situations. However, the broader physiological relevance of electrolyte concentration emerges when considering how hydration strategies, fitness level and heat acclimatisation shape sweat composition and thermoregulatory efficiency.

In individuals who are heat‐acclimatised or physically trained, sweat tends to be more dilute, containing lower concentrations of sodium and chloride in comparison to non‐acclimatised individuals (Klous et al., 2020). This dilution allows for slightly more efficient evaporation by minimizing the reduction in vapour pressure caused by dissolved ions. Conversely, during intense exertion, sweat can become progressively more concentrated, particularly if dehydration reduces total body water volume (Baker et al., 2019). Although the absolute effect of increased salinity on evaporation rate is small in comparison to factors such as humidity and airflow, it might contribute to reduced evaporative efficiency during extreme endurance events or military operations, as solutes accumulate on the skin over time owing to preferential water loss. Although the sweat being secreted might have a stable concentration, the residual sweat film becomes increasingly concentrated as water evaporates. Mathematically, the higher salinity reduces the probability that a molecule has sufficient energy to evaporate [$P(E\geq E_{\mathrm{vap}})$]. Although the primary purpose of reduced electrolyte loss is to preserve fluid and electrolyte balance, it also has a minor secondary role in supporting heat dissipation.

In addition to endogenous sweat evaporation, external application of water to the skin, such as dousing during exercise, is a widely used strategy to enhance cooling (Anderson et al., 2024). However, the effectiveness of dousing depends on the purity of the water applied. Pure or low‐solute water provides the best evaporative potential by maintaining a high vapour pressure at the skin interface and allowing efficient molecular escape into the air. In contrast, water contaminated with salts, minerals or impurities, such as from rivers, stagnant sources or seawater, has a reduced vapour pressure owing to the same ion–dipole interactions that lower the evaporation rate of salty sweat (Urbic, 2014). As a result, impure water might evaporate less efficiently, potentially slowing the rate of evaporative cooling (Yan et al., 2021). However, prior estimates indicate that the reduction in the heat of evaporation owing to typical sweat solutes is minimal, ∼0.014% (Wenger, 1972). Consequently, the evaporation of solute‐laden water remains highly effective, with emergency medicine guidelines emphasizing the importance of using any available water source for cooling (Pryor et al., 2023). Even in cases of extremely saline or contaminated water, significant cooling still occurs through both evaporation and conductive heat transfer owing to the high specific heat capacity of water, as seen in a variety of animals that do not produce sweat (Ingram, 1965). The concentration of impurities required to impede evaporation meaningfully in field conditions has yet to be clearly established. However, as impurity levels rise, the mixture will eventually reach a threshold where it behaves more like mud than water. In this state, the cooling mechanism shifts from evaporative to primarily conductive, with the mud absorbing heat from the body. Although some moisture within the mud might still evaporate and provide limited cooling, once the outer layer dries it functions as an insulator, trapping heat and impeding further heat loss (Tian et al., 2022).

### 6.3 Blood flow and skin temperature

Although sweat evaporation is the final pathway for removal of heat from the body, its effectiveness depends crucially on the rate at which heat is delivered to the skin surface. The skin can gain thermal energy through its own metabolism, direct solar radiation or heat exchange with a hot environment (Cramer & Jay, 2019). However, in most conditions, the majority of heat is transferred from the body core and passes through the skin to reach the sweat film. In this role, the skin and surrounding extravascular tissue act primarily as a conduit through which thermal energy is delivered to the surface for evaporative loss (Charkoudian, 2003). This heat delivery occurs via increased skin blood flow, driven by cutaneous vasodilatation during exercise or heat exposure. At rest, skin blood flow can be relatively low (∼0.25 L min^−1^), but during thermoregulatory stress it can increase to ∼8 L min^−1^ (Charkoudian, 2003). By maintaining a steep temperature gradient between the interior of the body and the skin, blood flow increases skin temperature and ensures that sufficient thermal energy is delivered through the surrounding tissues to the skin surface, where it can be absorbed by sweat during evaporation. This, in turn, increases the proportion of sweat molecules with enough kinetic energy to overcome the latent heat of vaporisation [$P(E\geq E_{\mathrm{vap}})$], thereby supporting net evaporative heat loss ($Q_{\mathrm{evap}}$). Without this upstream heat delivery, even abundant sweating would fail to cool the body effectively.

Skin blood flow is not static; it is highly responsive to both internal and external conditions, and its effectiveness as a thermoregulatory mechanism can be impaired significantly. Dehydration, for example, reduces plasma volume and lowers central venous pressure, activating baroreflex‐mediated vasoconstriction that limits the ability of the body to maintain high skin perfusion during heat stress (Charkoudian, 2003). Likewise, certain medications or autonomic dysfunctions can blunt the vasodilatory response, reducing heat transfer to the skin (Hospers et al., 2024). Even behavioural cooling strategies, such as applying cold packs or immersing limbs in cool water, can constrict skin vessels if the temperature stimulus is too strong, impairing heat delivery despite a cooler surface (Alba et al., 2019). On the contrary, fitness, younger age and heat acclimatisation all enhance cutaneous vascular responses (Tew et al., 2012). Acclimatised individuals often maintain higher skin blood flow at a given core temperature, promoting earlier and more sustained heat delivery to the periphery (Roberts et al., 1977). These physiological differences mean that two individuals with the same sweat rate might experience markedly different cooling effectiveness, depending on how well their cardiovascular system supports the evaporation process.

### 6.4 Humidity and air flow

Environmental conditions set the boundary for how effectively sweat can evaporate from the skin. The key driver is the vapour pressure gradient between the liquid sweat film and the surrounding air. In dry environments, this gradient is large, and evaporation proceeds rapidly (Che Muhamed et al., 2016). However, when the air is already saturated with moisture (i.e., high humidity), the gradient can become inhibitive, and sweat can simply drip off without evaporating (Gagnon & Crandall, 2018). In such conditions, despite high sweat rates, minimal heat is lost (Che Muhamed et al., 2016).

Although humidity does not change the amount of energy that a sweat molecule needs to evaporate, it reduces the likelihood that the molecule will remain in the vapour phase (Bond & Struchtrup, 2004). In humid air, the surrounding space is already saturated with water vapour, diminishing the vapour pressure gradient and increasing the chance that evaporated molecules will recondense (Bond & Struchtrup, 2004). As a result, evaporation slows not because molecules cannot escape, but because they are more likely to return. Understanding the environmental constraints on this process is essential for predicting and managing heat stress across a wide range of settings, from athletic performance to occupational safety.

Air movement plays a crucial supporting role in maintaining effective evaporative cooling (Cramer & Jay, 2019). In still, stagnant environments, water vapour from evaporated sweat accumulates in a thin layer immediately above the skin, known as the boundary layer (Taylor & Machado‐Moreira, 2013). As this layer becomes saturated with moisture, the local vapour pressure rises, reducing the gradient needed for continued evaporation (Taylor & Machado‐Moreira, 2013). Airflow enables the continuous removal of this humid boundary layer, preserving the vapour pressure gradient required for ongoing evaporation. Even mild airflow helps to sustain this process. Without removal of humid air, sweat molecules have nowhere to disperse, and further evaporation becomes progressively inhibited.

Clothing, too, can either aid or hinder this process. Loose, breathable fabrics promote airflow across the skin, while moisture‐wicking materials help to maintain a thin, continuous layer of sweat that maximises evaporative efficiency (Gavin, 2003). In contrast, impermeable or tightly sealed garments, such as those worn in industrial or protective settings, trap heat and moisture, effectively shutting down evaporation unless active cooling systems or ventilated designs are incorporated (Ashworth et al., 2020; Gavin, 2003).

Using the $f_{\mathrm{escape}}$ component from the previous equations, this can be broken down further into the environmental factors discussed above; $f_{\mathrm{escape}}$ can be considered a product of three multiplicative modifiers:

$$f_{\mathrm{escape}}=f_{\mathrm{humidity}}\times f_{\mathrm{airflow}}\times f_{\mathrm{clothing}}$$

Each factor ranges from zero (fully inhibited) to one (fully permissive). For humidity, higher relative humidity reduces the vapour pressure gradient and increases recondensation, leading to a lower $f_{\mathrm{humidity}}$, which can be approximated as:

$$f_{\mathrm{humidity}}\approx \left(1-\mathrm{RH}\right)\times \left(1-S\right)$$

where RH is the relative humidity expressed as a decimal, and S is the salinity factor, which reflects the impact of dissolved electrolytes on vapour pressure. For typical sweat, S is approximated as 0.00014 (Wenger, 1972). For $f_{\mathrm{airflow}}$, high velocities correspond to values closer to one, whereas stagnant air approaches zero, although natural convection ensures some minimum level of air movement. For $f_{\mathrm{clothing}}$, loose, breathable garments would be represented by values near one, whereas impermeable or tight‐fitting clothing would have values closer to zero. Together, these constituents of $f_{\mathrm{escape}}$ directly determine the fraction of surface molecules that contribute to the net evaporative heat loss ($Q_{\mathrm{evap}}$).

## 7 IMPLICATIONS FOR HUMAN THERMOREGULATION

The molecular understanding of sweat evaporation carries important implications for exercise physiology, occupational performance and the management of heat stress. It reframes cooling efficiency as not merely a function of how much sweat is produced, but of how effectively internal and external conditions support the phase change process at the skin surface. This perspective helps to explain individual variability in heat tolerance, whereby two people with similar sweat rates can experience vastly different thermal strain depending on acclimatisation status, blood flow or environmental constraints (Cramer & Jay, 2019; Gagnon & Crandall, 2018; Roberts et al., 1977).

For practitioners, it highlights the importance of supporting not only hydration and electrolyte replacement, but the full evaporative process. Strategies such as ensuring adequate airflow, choosing breathable clothing and pacing activity to preserve skin wettedness directly influence the molecular conditions that allow sweat to function as a cooling mechanism. It also clarifies why some common cooling interventions, such as misting, dousing or fan use, can be highly effective in dry environments but counterproductive in humid conditions.

Ultimately, integrating the molecular physics of evaporation into physiological thinking enhances the ability to anticipate, measure and support human performance and safety in the heat. As research advances, these insights could inform predictive models of heat strain, the design of wearable sensors, or adaptive interventions that account not only for sweat volume but also for how efficiently individual sweat droplets are doing the molecular work of cooling.

## 8 CONCLUSION

Evaporative cooling is often treated in physiological literature as a bulk surface phenomenon, but its effectiveness is rooted in molecular physics. The process hinges on individual water molecules gaining enough kinetic energy to escape the liquid phase, which is a probabilistic event governed by the Boltzmann distribution. This phase change is energetically costly, requiring a significant amount of latent heat drawn from the skin, which cools the body in the process. Although the production of sweat is essential, its thermoregulatory impact depends on far more than secretion rate alone. Internal factors, such as skin blood flow, electrolyte composition and sweat distribution, interact with external influences, such as humidity, airflow and clothing, to determine whether evaporative cooling occurs and how efficiently it proceeds. This culminates in the expanded equation:

$$Q_{\mathrm{evap}}\propto A_{\mathrm{skin}}\times \rho_{\mathrm{film}}\times \delta \times \times f_{\mathrm{humidity}}\times f_{\mathrm{airflow}}\times f_{\mathrm{clothing}}\times E_{\mathrm{loss}}$$

which unifies surface properties, molecular thermodynamics and environmental constraints into a single expression for evaporative heat loss.

Physiologists seeking to understand heat stress, thermoregulation and performance in the heat must consider these layers of control. A well‐hydrated, heat‐acclimatised individual in a dry, breezy environment can experience highly efficient cooling through a thin film of dilute sweat. In contrast, the same person in a hot, humid and still environment can experience ineffective evaporation (even with maximal sweating) simply because the external conditions limit the vapour pressure gradient. Understanding the underlying molecular and physical mechanisms provides clearer insight into why certain interventions work, why others fail, and how human physiology interfaces with environmental physics to regulate heat.

## AUTHOR CONTRIBUTIONS

Edward T. Ashworth conceived the review, conducted the literature analysis, wrote the manuscript and approved the final version.

## CONFLICT OF INTEREST

None declared.

## FUNDING INFORMATION

None.
